# Effectiveness of Probiotic, Prebiotic, and Synbiotic Supplementation to Improve Perinatal Mental Health in Mothers: A Systematic Review and Meta-Analysis

**DOI:** 10.3389/fpsyt.2021.622181

**Published:** 2021-04-22

**Authors:** Vidhi Desai, Anita L. Kozyrskyj, Stuart Lau, Omolara Sanni, Liz Dennett, Jens Walter, Maria B. Ospina

**Affiliations:** ^1^Department of Obstetrics & Gynecology, Faculty of Medicine and Dentistry, University of Alberta, Edmonton, AB, Canada; ^2^Department of Pediatrics, Faculty of Medicine and Dentistry, University of Alberta, Edmonton, AB, Canada; ^3^School of Public Health, University of Alberta, Edmonton, AB, Canada; ^4^Faculty of Science, McGill University, Montreal, QC, Canada; ^5^John W. Scott Health Sciences Library, University of Alberta, Edmonton, AB, Canada; ^6^Department of Medicine, School of Microbiology, University College Cork, National University of Ireland, Cork, Ireland; ^7^APC Microbiome Institute Ireland, University College Cork, National University of Ireland, Cork, Ireland

**Keywords:** pregnancy, postpartum depression, probiotics, prebiotics, synbiotics, mental health disorders, depression, anxiety

## Abstract

**Introduction:** There is an emerging interest in modulating the gut microbiota to target the gut-brain axis and improve maternal mental health in the perinatal period. This systematic review evaluated the effectiveness of prebiotics, probiotics, and synbiotics supplementation during pregnancy to reduce the risk of maternal mental health problems in the perinatal period.

**Methods:** Electronic biomedical databases and clinical trial registries were searched from database inception through August 2020 to identify randomized controlled clinical trials (RCTs) evaluating the effect of probiotic, prebiotic, or synbiotic supplements administered to women during pregnancy on measures of perinatal depression, anxiety, and other mental health outcomes. Study selection, risk of bias appraisal, and data extraction were independently performed by two reviewers. Pooled mean differences (MD) and odds ratios (pOR) with 95% confidence intervals (CI) were calculated in random-effects meta-analyses for the outcomes of interest in the review.

**Results:** From 3,868 studies identified through the search strategy, three RCTs of low risk of bias involving 713 participants were included, all three testing probiotics. There were no differences between probiotics and control groups in the mean depression scores (MD −0.46; 95% CI −2.16, 1.25) at end of follow-up. Although statistical significance was not achieved, probiotics showed an advantage in the proportion of participants scoring below an established cut-off for depression (pOR 0.68; 95% CI 0.43, 1.07). Compared to placebo, probiotics in pregnancy reduced anxiety symptoms (MD −0.99; 95% CI −1.80, −0.18); however, this advantage was not translated in a reduction in the proportion of participants scoring above an established cut-off for anxiety (pOR 0.65; 95% CI 0.23, 1.85). There were no differences between probiotics and control groups in global mental health scores at end of follow-up (MD 1.09; 95% CI −2.04, 4.22).

**Conclusion:** There is limited but promising evidence about the effectiveness of probiotics during pregnancy to reduce anxiety symptoms and reduce the proportion of women scoring ABOVE a cut-off depression score. There is a lack of RCT evidence supporting prebiotics and synbiotics supplementation for similar purposes in the perinatal period. More research is needed before prebiotics, probiotics, and synbiotics are recommended to support maternal mental health and well-being in the perinatal period.

**Systematic Review Registration:** PROSPERO, CRD42019137158.

## Introduction

Maternal mental health problems in the perinatal period are a global public health challenge. As many as one in five women develop depression (1) and/or anxiety (2) in the postpartum period, making them the most common complications of pregnancy and delivery (3). When untreated, these conditions can have devastating long-term effects on mothers, children, families, and society at large (4). Maternal mental health disorders are associated with an increased risk of low birthweight, premature birth, impaired mother–infant attachment, and infant malnutrition in the first year of life (4). Postpartum mental health problems impose a substantial economic burden on health care systems and society, costing as much as $150,000 per case over the lifespan (5).

Dysbiosis or altered community composition of gut microbiota is linked to the origins of an ever-expanding set of inflammatory and non-communicable diseases (6, 7). Our gut microbial composition varies by age, gender, diet and relevant to disease pathogenesis, early-life environmental exposures (6–9). There is growing evidence for the role of gut microbiota in the development and course of many mental health problems (10–12). The gut-brain axis is the bidirectional communication pathway between the enteric and central nervous systems (13). These interactions have been proposed as factors in the pathophysiology of major psychiatric disorders, such as schizophrenia (12, 14), and of depression and anxiety (15). Direct and indirect mechanisms involving potential roles for short chain fatty acids, bile acids, neurotransmitters, and other microbiota-derived products have been proposed to modulate central nervous system function and neuroinflammation (16, 17). The cytokines that are produced in the brain and the periphery as a result of the inflammation (18) influence neurotransmitter synthesis, release and reuptake (19). Findings from animal models have provided strong evidence for a causal role of gut microbiota in mental health problems (10).

Not surprisingly, there is an emerging interest in microbiome modification through the administration of probiotics, prebiotics, and synbiotics, to improve aspects of the gut-brain axis (20). Probiotics are live microorganisms that when ingested can improve the host's health or physiology (20). Prebiotics are non-digestive food ingredients that induce beneficial changes to gut microbiota composition; synbiotics are food ingredients and dietary supplements that contain both prebiotics and probiotics (20). A recent systematic review reported that probiotics were associated with reduced depressive symptoms in the general population (21, 22). Furthermore, daily probiotic supplementation is thought to have beneficial effects on mood, anxiety, and major depressive disorder cognitive symptoms (23). The evidence around prebiotics is not as concrete. Recent reviews concluded that prebiotic supplementation, either alone or combined with probiotics, can have beneficial effects on mental health disorders (24, 25). In contrast, another review reported that prebiotic supplementation does not improve depression or anxiety symptoms (22). There is a lack of evidence on the effect of synbiotic supplementation and mental health disorders.

Prebiotic, probiotic, and synbiotic supplements are increasingly been used during pregnancy to reduce the risk of maternal mental health disorders during the perinatal period, but their effects have not been systematically evaluated. The objective of our systematic review was to synthesize this emerging literature, namely to evaluate the evidence on the administration of prebiotic, probiotic, and/or synbiotic supplements during pregnancy to reduce the risk of mental health problems in the perinatal period.

## Materials and Methods

This systematic review was reported and conducted in accordance to Preferred Reporting Items for Systematic Reviews and Meta-Analyses (PRISMA) statement (26). A protocol for this review was registered in the International prospective register of systematic reviews (PROSPERO 2019 CRD42019137158).

### Search Strategy

Comprehensive literature searches of biomedical electronic databases [MEDLINE (Ovid interface), EMBASE (Ovid interface), CINAHL Plus with Full Text (EBSCOhost interface), Cochrane Central Register of Controlled Trials (Wiley interface, which also includes ClinicalTrials.gov and the WHO International Clinical Trials Registry Platform), Scopus, Web of Science Core Collection, and BIOSIS (Web of Science Platform)] were conducted from database inception to August 19, 2020. The search strategy was designed by a health sciences librarian (LD) and comprised of both selected subject headings and free terms related to probiotics (i.e., living microorganisms such as *Bifidobacterium, Lactobacillus, Saccharomyces, Lactococcus, Bacillus*), prebiotics (such as fructooligosaccharides, galactooligosaccharides, and xylooligosaccharides), and their combination (synbiotics) and then combined with terms for the concept of pregnancy. The search was limited to randomized controlled trials (RCTs) using the Glanville et al. (27) RCT filter and animal studies were removed where possible. No language or date limits were applied. Conference abstracts were retrieved in Embase and Web of Science. Details of the search strategy used for MEDLINE are included in Supplementary Material 1 (search strategies for other databases are available upon request). Reference lists of potentially relevant articles were reviewed, and additional web searches were conducted to identify studies that were not identified through literature searching. No language or publication status restrictions were applied to the literature searches.

### Study Eligibility Criteria

Parallel randomized controlled trials (RCTs) evaluating the administration of prebiotic, probiotic, and synbiotic supplementation during pregnancy were considered for inclusion in the review. Studies should have been conducted in clinical (prenatal care) settings or in the general population and included populations of women with uncomplicated pregnancies (i.e., no gestational hypertension, preeclampsia, and gestational diabetes). The intervention of interest was probiotic, prebiotic, or synbiotic supplements, alone or combined, administered orally to women anytime during pregnancy, at any dose, with the intention to treat for a minimum of seven days. Primary outcomes in the review were measures of maternal mental health (i.e., depression and anxiety and other mental health problems) anytime during pregnancy (after trial enrollment) and/or in the first 12 months after delivery, expressed proportions of women with a diagnosis of mental health disorder, or changes in scores from baseline for questionnaire data. Studies that reported outcome measures using structured clinical interviews (e.g., Clinical Interview Schedule-Revised) or validated screening questionnaires (e.g., Edinburgh Postnatal Depression Scale, Kessler Psychological Distress Scale) were considered for inclusion. Excluded from the review were review articles, editorials, letters, case series, case reports, quasi-experimental studies, other observational studies, and cross-over trials.

Two pairs of independent reviewers (VD and SL, and VD and MO) screened titles and abstracts generated from the searches to identify potentially relevant studies. The full text of articles deemed relevant and those whose abstracts and titles provided insufficient information were retrieved and independently assessed for eligibility in the review. Disagreements about study eligibility were resolved through discussions among reviewers until a consensus was reached.

### Risk of Bias Assessment

Two pairs of independent reviewers (VD and OS, and VD and MO) assessed the risk of bias of primary studies using standardized instruments based on study design. Discrepancies in risk of bias assessment were resolved through discussions among reviewers until consensus was reached Risk of bias of RCTs was assessed using the Cochrane Risk of Bias (RoB) tool, which included the following critera: random sequence generation, allocation concealment, blinding of participants and personnel, bliding of outcome assessment, incomplete outcome data, selective reporting and other sources of bias (28). For each criteria, the studies were either judged as meeting (low risk of bias) or not meeting (high risk of bias) the criteria based on predetermined guidelines. Then, an overall assesment of RoB (high, moderate, low) was assigned to the individual studies based on Cochrane guidelines for summarizing risk of bias across bias domains (28).

### Data Extraction

The following information was extracted from primary studies using a data extraction form: year, country, study design, characteristics of the population, intervention and comparison groups, and study outcomes. Data from the included studies was extracted by one reviewer (VD) and then independently verified for accuracy and completeness by a second reviewer (OS or MO). Discrepancies in data extraction were resolved by a consensus.

### Synthesis of the Results and Grading of the Evidence

A narrative synthesis of outcomes across the studies was undertaken and summary tables were used to describe the characteristics of the populations, interventions, comparison groups, and outcomes of primary studies included in the review. Meta-analyses were planned if there were at least two sufficiently homogenous studies reporting on the outcome of interest (i.e., similar study population, intervention and reported outcomes). We conducted meta-analyses of RCT data using a random effects model. Pooled odds ratios (pOR) for categorical data and mean differences (MD) for continuous outcome data (when outcomes at the end of follow-up were reported using the same measurement instrument) were reported with 95% confidence intervals (CI) around the effect estimates. Forest plots were used to display individual and pooled trial results. Statistical heterogeneity across trials was quantified using the *I*^2^ statistic (29). Heterogeneity of effect estimates across trials was described as small (*I*^2^ < 25%), moderate (*I*^2^ between 26 and 74%) or (*I*^2^ ≥ 75%) (29). We explored sources of heterogeneity qualitatively by comparing study designs, exposures assessed, maternal mental and health outcomes studied. Publication bias was to be assessed using funnel plots (30), where at least 10 RCTs were available from the meta-analyses. We planned subgroup analyses by probiotic, prebiotic, or synbiotic types/doses and also by pregnancy and postpartum periods in which outcomes occurred. Subgroup analyses by relevant demographic or clinical characteristics (baseline nutritional status/diet, and ethnicity) were also considered. Study selection, quality assessment, and data extraction was managed with Microsoft Excel™ (Microsoft Corporation, Redmond, WA). Statistical analyses were performed using Review Manager (RevMan) version 5.3 (Copenhagen: The Nordic Cochrane Center; The Cochrane Collaboration 2014).

The quality of the evidence for each outcome in the review that included pooled RCT data was assessed using the Grading of Recommendations Assessment, Development and Evaluation (GRADE) framework (31). The GRADE framework uses four domains to downgrade RCT evidence based on (1) study limitations (risk of bias), (2) indirectness of evidence, (3) inconsistency in the results, and (4) imprecision of effect estimates or potential publication bias. The GRADE assessment was conducted independently by two reviewers (VD and MO). Disagreements were resolved by consensus. The GRADE approach was used to interpret the findings (32). An overall strength of evidence rating was assigned for each outcome reported in the review as follows: High quality (i.e., further research is very unlikely to change our confidence in the estimate of effect); Moderate quality (i.e., further research is likely to have an important impact on our confidence in the estimate of effect and may change the estimate), Low quality (i.e., further research is very likely to have an important impact on our confidence in the estimate of effect and is likely to change the estimate), and Very low quality (i.e., we are very uncertain about the estimate) (32). Results were presented in a summary of findings table.

## Results

### Search Results

**Figure 1:**
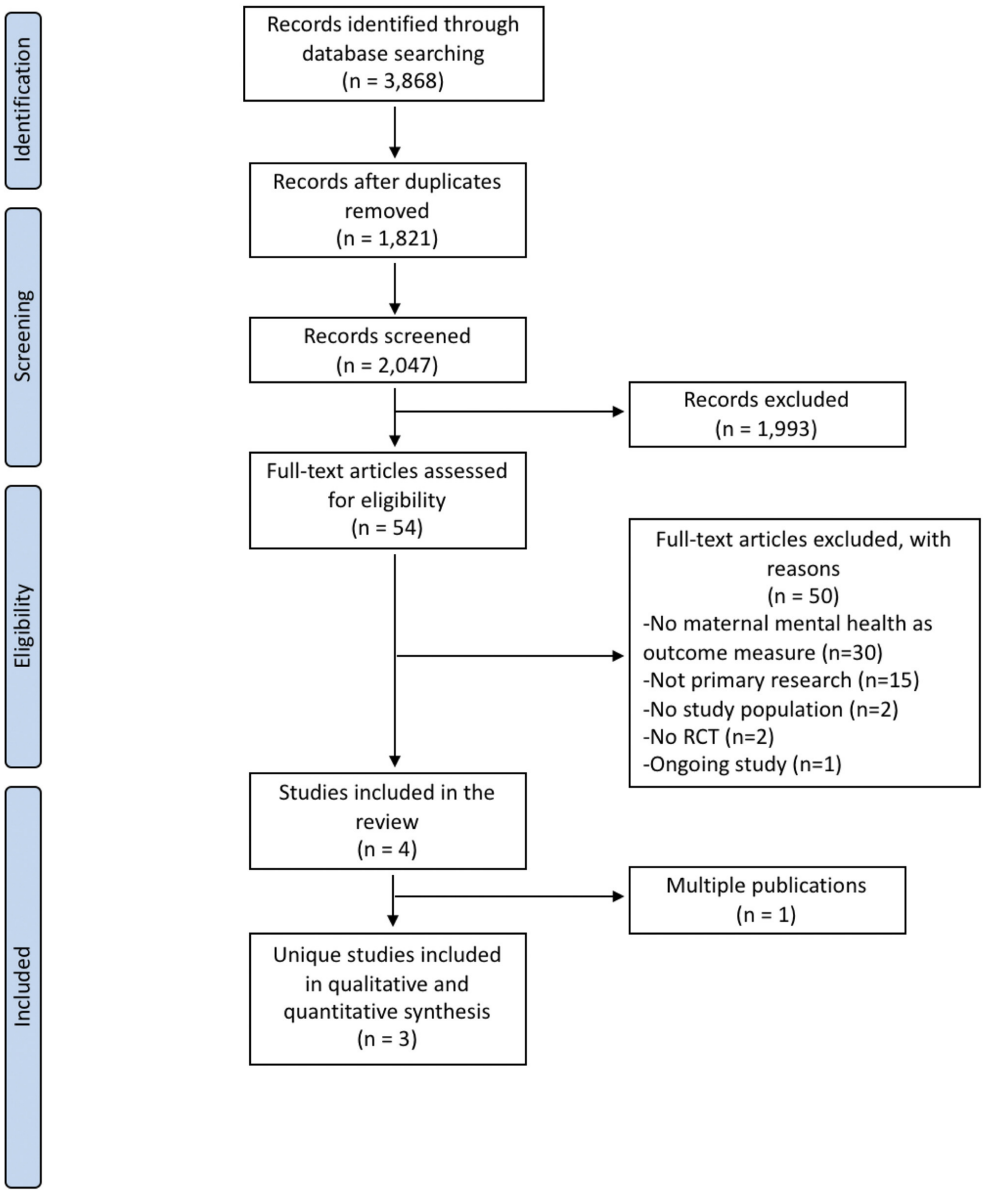
PRISMA flow diagram of search strategy and study selection.

Electronic and gray literature searches identified a total of 3,868 potentially relevant citations. After removal of 1,821 duplicates, titles and abstracts of 2,047 references were screened. At this stage, 54 full-text articles were judged to be potentially relevant, of which 4 articles satisfied the review eligibility criteria (33–36). Of these, one reference (35) was a multiple publication of another trial (33) and, therefore, the systematic review included 3 unique studies (33, 34, 36) reported in 4 publications. The study selection flow diagram is presented in Figure 1. The remaining 50 full-text records were excluded for the following reasons: maternal mental health was not an outcome measure (*n* = 30), not primary research (*n* = 15), incorrect study population (*n* = 2), no RCT design (*n* = 2), and ongoing study (*n* = 1). The complete references of excluded studies and reasons for exclusion is available upon request. One ongoing study (37) was identified as potentially relevant; however, when contacted, the authors were unable to provide data that could be included in this systematic review.

### Characteristics of Included Studies

The effectiveness of pregnancy use of probiotics, probiotics, and synbiotics to reduce the risk of mental health problems in the perinatal period is a relatively new area of research, with three trials published between 2016 and 2020. Studies were conducted in New Zealand (33, 36) and Iran (34). Studies were funded by university (34), and research grants and industry partners (33, 36). One study was conducted in health care settings (34), while the other two (33, 36) were conducted among pregnant women in the community. Study populations in the trials were diverse and included constipated pregnant women between 24 and 28 weeks gestational age (34), healthy pregnant women between 14 and 16 weeks gestational age (36), and obese [body mass index (BMI) ≥30 kg/m^2^] pregnant women between 12 and 17 + 6 weeks gestational age (33). The age distribution of participants across trials was similar, with mean ages ranging from 28.6 (34) to 33.5 years (36). Two trials (33, 36) described diverse ethnic composition in their study samples, while one trial did not report the ethnicities represented in the study (34). The three trials (33, 34, 36) reported socioeconomic characteristics of the study populations using different categories of income and education. Overall, over 50% of participants in the trials had at least post-secondary educational background and medium-to-high income levels; however, the distribution of these characteristics in the study population were heterogeneous across the studies.

The three RCTs evaluated probiotic supplementation administered in capsules (33, 36) or as yogurt-enriched formula (34). None of the trials evaluated the administration of prebiotic or synbiotic supplements during pregnancy. Among the trials that administered probiotic supplementation in capsules, one (36) administered one probiotic capsule daily, containing *Lactobacillus rhamnosus* HN001 at a dose of 6 × 10^9^ colony-forming units (cfu), from enrollment until 6 months after birth, if breastfeeding, or before if the participants did not breastfeed for 24 h. The other trial (33) administered one probiotic capsule daily, containing *Lactobacillus rhamnosus* GG and *Bifidobacterium lactis* BB12 at a dose of 6.5 × 10^9^ cfu, from enrollment until birth. Both trials used placebo as comparators. The trial that used a probiotic-enriched yogurt (34), administered 300 grams of probiotic yogurt, enriched with 4.8 × 10^10^ CFU of *Lactobacillus acidophilous* and *Bifidobacterium lactis*, 3 times per day for 4 weeks. The control group received conventional yogurt at similar dose and frequency of administration.

Outcomes evaluated in RCTs included measures of depression and anxiety symptoms, expressed as symptom scores at follow-up, and as proportion of participants who scored at or above pre-specified cut-off points for potential depression or anxiety (33, 36). The studies used the Edinburgh Postnatal Depression Scale (EPDS) (38) and the State Trait Anxiety Inventory 6 item version (STAI-6) (39) to evaluate depression and anxiety outcomes, respectively. RCTs (33, 34) also reported mean scores at follow-up on mental health subscales of the SF-36 quality of life instrument, where lower scores indicate more disability (40). Other mental health outcomes were not examined in the primary studies. Detailed characteristics of the individual studies are presented in Table 1.

**Table 1:** Characteristics of the included studies.

**Study**	**Trial characteristics**	**Population characteristics**	**Intervention characteristics**	**Intervention and comparison groups**	**Outcomes and results**		
Dawe et al. and Okesene-Gafa et al. (33, 35) New Zealand **Funding:** Research grants, Industry	Parallel RCT **Enrollment:** Apr 2015–Jun 2017 **Setting:** Community	*N* = 230 **Clinical characteristics:** Pregnant women BMI ≥30 kg/m^2^ **Mean age (SD) (yr):** 29.7 (5.4). Intervention: 30.0 (5.5); Placebo: 29.39 (5.3) **Gestational age:** 12–17 (+6 days) wks **Ethnicity:** Maori (20%), Pasifika (48%), Asian (9%), Latin American/African (2%), European (21%) **SES characteristics:** **Income:** Highest deprivation quintile (63%) **Education:** Incomplete high school (28%), complete high school (15%), diploma (20%); tertiary (37%)	**Intervention:** Probiotic (capsules) Lactobacillus rhamnosus GG and Bifidobacterium lactis BB. 12 6.5 × 10^9^ cfu. 1 dose per day **Control:** Placebo capsules (microcrystalline cellulose and dextrose anhydrate) **Period of initiation:** End of 1st trimester (12–17 + 6 d wks) **Duration:** From 12–17 (+6 days) wks−36 wks of pregnancy	**Intervention** *N* randomized = 115 *N* analyzed = 88 *N* dropouts = 27 **Control** *N* randomized = 115 *N* analyzed = 76 *N* dropouts = 39	Period of outcomes assessment: 36 wks gestation		
					Depression: EPDS depression scores		
						Mean score, SD	
					Intervention (*n* = 88)	7.2 (3.8)	
					Control (*n* = 76)	6.7 (4.6)	
					Depression (% scoring at cut-off ≥13 for depression)		
						Yes	No
					Intervention (*n* = 88)	8 (9%)	80 (91%)
					Control (*n* = 76)	8 (11%)	68 (89%)
					Anxiety: STAI-6 anxiety scores		
						Mean score, SD	
					Intervention (*n* = 88)	31.9 (10.2)	
					Control (*n* = 76)	32.8 (10.3)	
					Anxiety (% scoring at or above >15 cut-off in STAI-6) (35)		
						Yes	No
					Intervention (*n* = 87)	6 (6.9%)	81 (93.1%)
					Control (*n* = 77)	4 (5.2%)	73 (94.8%)
					Mental health: SF-36 Mental Health subscale scores		
						Mean score, SD	
					Intervention (*n* = 88)	48.6 (8.5)	
					Control (*n* = 76)	48.3 (9.8)	
Mirghafourvand et al. (34) Iran **Funding:** University	Parallel RCT **Enrollment:** Dec 2014–Jul 2015 **Setting:** Health care centers	*N* = 60 **Clinical characteristics:** Pregnant women; Constipation (ROME III criteria **Mean age (SD) (yr):** 28.6 (NR) Intervention: 28.5 (NR) Control: 28.77 (NR) **Gestational age:** 24–28 wks **Ethnicity:** NR	**Intervention:** Probiotic (yogurt) Bifidobacterium lactis and Lactobacillus acidophilus 4.8 × 10^10^ cfu. 300 gr 3x/day. **Control:** Conventional yogurt. 300 gr 3x/day. **Period of initiation:** End of 2nd trimester (24–28 wks) **Duration:** 4 wks	**Intervention** *N* randomized = 30 *N* analyzed = 29 *N* dropouts = 1 **Control** *N* randomized = 30 *N* analyzed = 28 *N* dropouts = 2	Period of outcomes assessment: 30–34 wks gestation Mental health: SF-36 Mental Health subscale scores		
						Mean score, SD	
					Intervention (*n* = 30)	60.1 (10.7)	
					Control (*n* = 30)	55.7 (16.1)	
		**SES characteristics:** **Income:** Favorable (10%); partly favorable (76.7%), unfavorable (13.3%) **Education:** Primary (23.3%), secondary (10%), diploma (45%), university (21.7%)					
Slykerman et al. (36) New Zealand **Funding:** Research grants, Industry	Parallel RCT **Enrollment:** Dec 2012–Nov 2014 **Setting:** Community	*N* = 423 **Clinical characteristics:** Pregnant women; healthy **Mean age, SD (yr):** 33.5 (4.3) Intervention: 33.5 (4.2) Control: 33.7 (4.4) **Gestational age:** 14–16 wks **Ethnicity:** Maori (12.9%); Pacific (2.1%); Asian (7.1%); European (77.6%); Other (0.3%) **SES characteristics:** **Income:** 0–49k (6.6%); 50–99k (32.1%), 100–149k (35.5%); 150+k (25.8%) **Education:** School (12.6%), post school (11.3%), university (76.1%)	**Intervention:** Probiotic (capsules) Lactobacillus rhamnosus HN001; Capsules (6 × 10^9^ cfu/day) **Control:** Placebo capsules (corn-derived maltodextrin). **Period of initiation:** Start of 2nd trimester (14–16 wks) **Duration:** From 14–16 wks to 6 mo postpartum	**Intervention** *N* randomized = 212 *N* analyzed = 193 *N* dropouts = 19 **Control** *N* randomized = 211 *N* analyzed = 187 *N* dropouts = 24	Period of outcomes assessment: 12 mo postpartum Depression: EPDS depression scores.		
						Mean score, SD	
					Intervention (*n* = 194)	7.7 (5.4)	
					Control (*n* = 187)	9.0 (6.0)	
					Depression (% scoring at or above >12 cut-off in EPDS)		
						Yes	No
					Intervention (*n* = 194)	32 (16.5%)	162 (83.5%)
					Control (*n* = 187)	44 (23.5%)	143 (76.5%)
					Anxiety: STAI-6 anxiety scores		
						Mean score, SD	
					Intervention (*n* = 192)	12.0 (4.0)	
					Control (*n* = 187)	13.0 (4.3)	
					Anxiety (% scoring at or above >15 cut-off in STAI-6)		
						Yes	No
					Intervention (*n* = 192)	30 (15.6%)	162 (84.4%)
					Control (*n* = 187)	55 (29.4%)	132 (70.6%)

*BMI, body mass index; cfu, colony forming units; EPDS, Edinburgh Postnatal Depression Scale; gr, grams; mo, months; NR, not reported; RCT, randomized controlled trial; SD, standard deviation; SES, socioeconomic status; STAI-6, State Trait Anxiety Inventory 6 item version; wk, weeks; yr, years*.

### Risk of Bias Assessment

**Figure 2:**
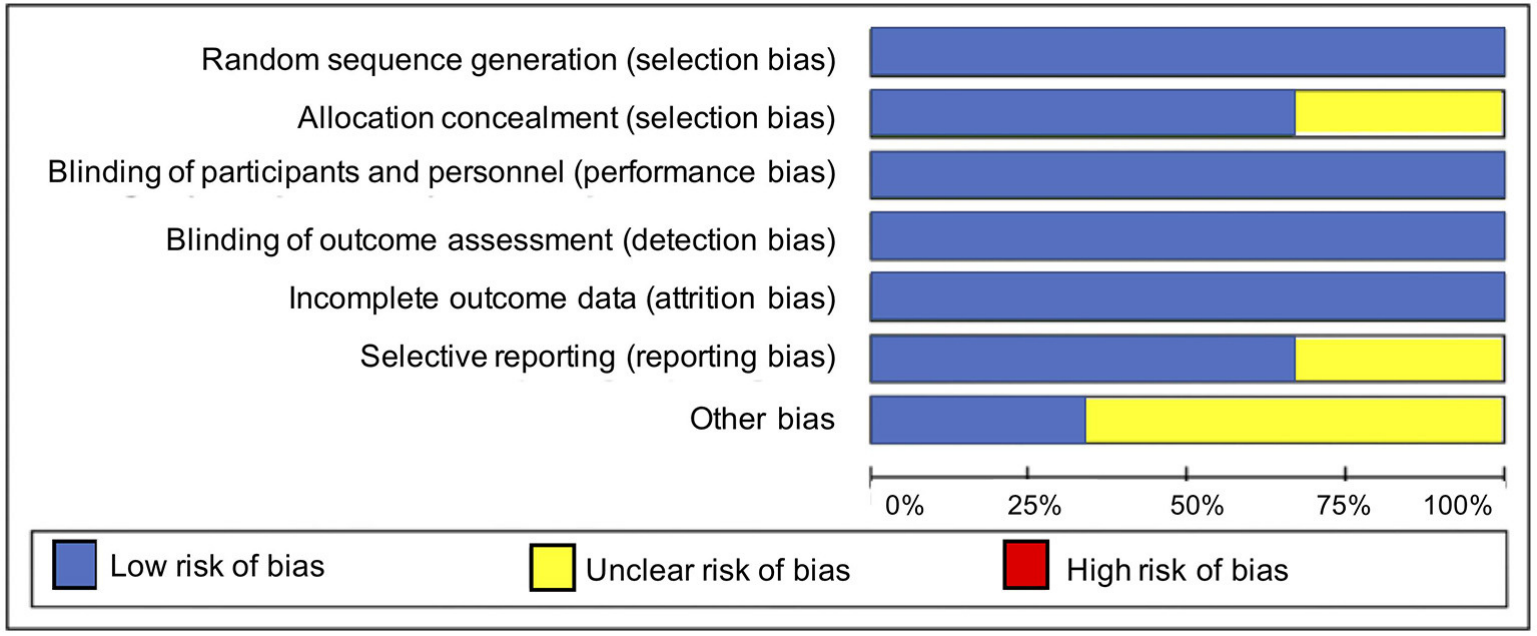
Risk of bias graph.

**Figure 3:**
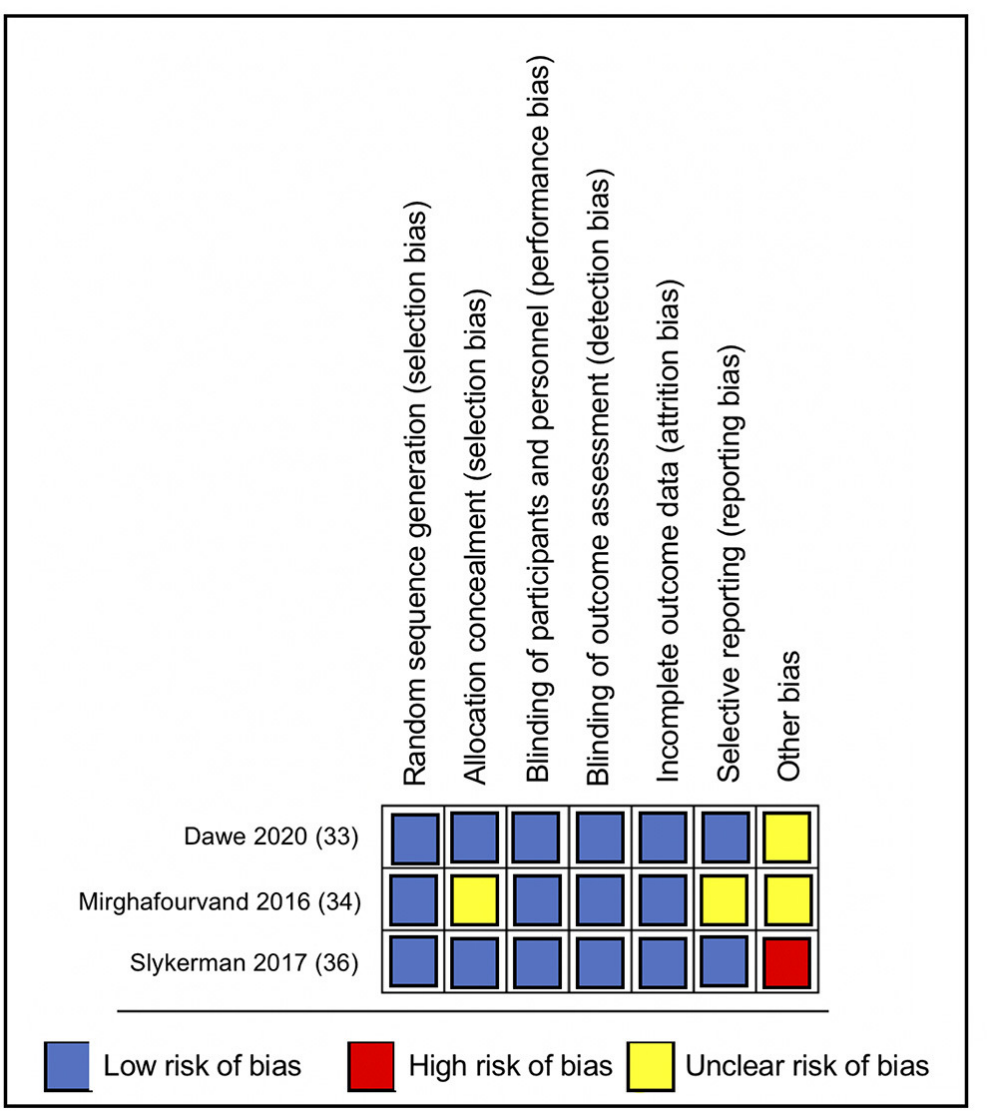
Risk of bias summary.

Overall, the included studies had a low risk of bias for most bias domains, except for the domain of other sources of bias, in which trials were rated as unclear (33, 34) or at high risk of bias (36) (Figure 2). In the RCT conducted by Mirghafourvand et al. (34) the description of the allocation concealment was unclear, and the probiotic and conventional yogurt was provided by Pegah Dairy Industries Co. in Tabriz, Iran. However, the authors did not explicitly state the level of involvement Pegah Dairy Industries had in the research study. The probiotic and placebo capsules in the RCTs conducted by Slykerman et al. (36) and Dawe et al. (33) were also provided by industry, Fonterra Co-operative Group Ltd. and Chr. Hansen, respectively. Albeit the random allocation and concealment of treatment in the Slykerman et al. (36) trial were well-described, they were conducted centralized by the industry partner. Blinding of study participants, personnel and outcome assessment were appropriate across trials (Figure 3).

The three RCTs (33, 34, 36) contributed with follow-up data from 713 participants for meta-analyses comparing the effects of probiotics vs. control groups for the following outcomes: depression [mean EPDS depression scores at the end of follow-up and percentage of participants scoring above a cut-off score in the EPDS (33, 36)], anxiety (mean STAI-6 anxiety scores at the end of follow-up and percentage of participants scoring above a cut-off score in the STAI-6) (33, 36)and global measures of mental health [mean SF-36 mental health scores at follow-up (33, 34)].

### Depression Outcomes

**Figure 4:**
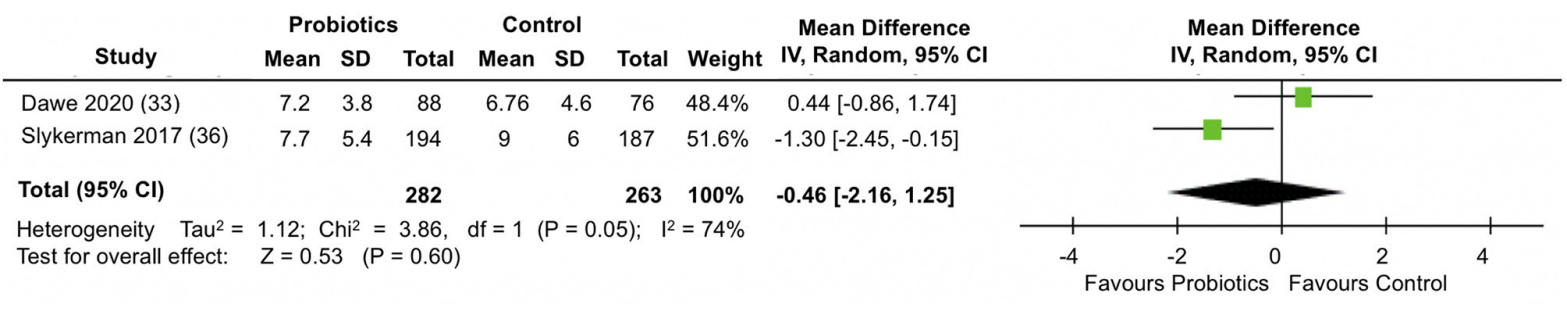
Meta-analysis of the effect of probiotic supplementation on EPDS depression scores in the perinatal period.

A meta-analysis of two trials (33, 36) involving a total of 545 participants (Figure 4) showed no differences between the probiotic and control groups in the mean EPDS depression scores at follow-up (MD = −0.46; 95% CI −2.16, 1.25; *I*^2^ = 74%). The results were heterogeneous across the trials. Potential sources of heterogeneity include differences in study populations. While the Slykerman et al. (36) trial included women with healthy pregnancies, participants in the Dawe et al. (33) trial had BMI indicative of obesity. Although the probiotic doses were similar in the two trials, there were differences in the timing and duration of the intervention. The Dawe et al. (33) trial administered the probiotics intervention from 12 to 17 (+6 days) weeks until 36 weeks of pregnancy, while the Slykerman et al. (36) trial commenced probiotic treatment around the same gestation and extended it to 6 months postpartum.

**Figure 5:**
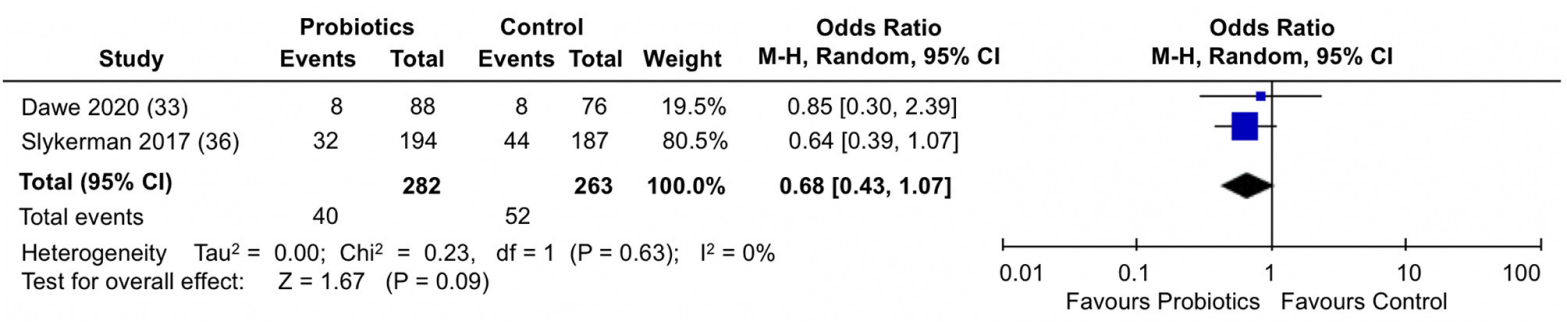
Meta-analysis of the effect of probiotic supplementation on the proportion of participants scoring above cut-off in EPDS depression scores in the perinatal period.

When the percentage of participants scoring above a cut-off score in the EPDS were analyzed in the two trials (33, 36) (Figure 5), participants in the probiotics groups showed a slight, but not statistically significantly reduction in the proportion of participants scoring above a cut-off score for depression in the EPDS (pooled OR: 0.68; 95% CI 0.43, 1.07; *I*^2^ = 0%). Results across the trials were homogeneous.

### Anxiety Outcomes

**Figure 6:**
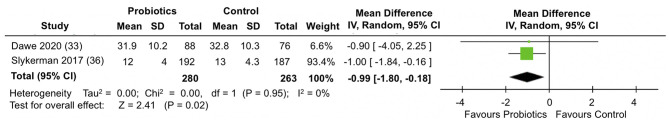
Meta-analysis of the effect of probiotic supplementation on STAI-6 anxiety scores in the perinatal period.

A meta-analysis of two RCTs (33, 36) involving data from 543 participants (Figure 6) showed that compared to placebo, probiotics administration during pregnancy significantly reduced anxiety scores in the STAI-6 questionnaire by almost 1 point at the end of follow-up (MD: −0.99; 95% CI −1.80, −0.18; *I*^2^ = 0%). Results were homogeneous across the trials.

**Figure 7:**
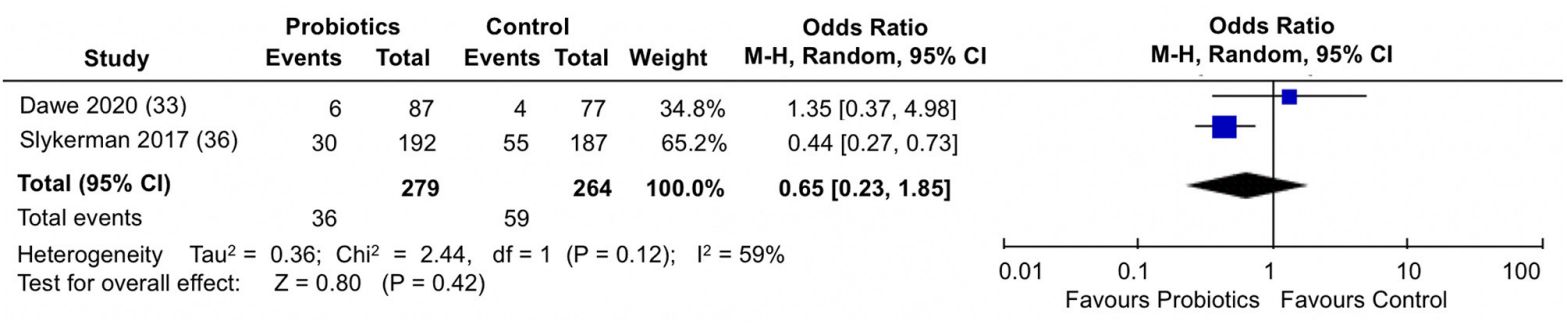
Meta-analysis of the effect of probiotic supplementation on the proportion of participants scoring above cut-off in STAI-6 anxiety scores in the perinatal period.

When the proportion of participants scoring above a cut-off in STAI-6 anxiety scores were analyzed (Figure 7), the pooled estimate from the two trials (33, 36) showed no differences between probiotics and control groups (pooled OR: 0.65; 95% 0.23, 1.85; *I*^2^ = 59%).

### Overall Mental Health Outcomes

**Figure 8:**
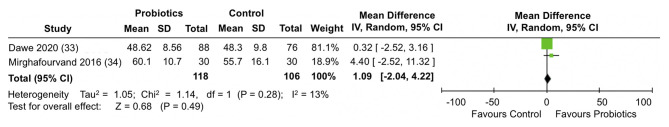
Meta-analysis of the effect of probiotic supplementation on SF-36 mental health scores in the perinatal period.

A meta-analysis of two trials (33, 34) involving a total of 224 participants (Figure 8) did not show differences between the probiotic and control groups in the mean SF-36 mental health scores at the end of follow-up (MD = 1.09; 95% CI −2.04, 4.22; *I*^2^ = 13%).

A formal evaluation of publication bias was not feasible as only 3 trials were identified in the review. Similarly, the small number of studies assessing the outcomes of interest in the review precluded subgroup analyses by relevant characteristics of the populations and the interventions under study.

### Quality of the Evidence

The quality of evidence was rated as moderate to low across the different outcomes (Table 2). The main limiting factor, which was the reason for a decrease in quality of the evidence for some outcomes, was the inconsistency of results across the small number of studies included in the meta-analyses. With only two studies included per meta-analysis, it is important to acknowledge the large potential impact if the average effect of one study differs in size and direction. The high heterogeneity in the EPDS depression scores in the primary trials and the moderate heterogeneity for the proportion of participants scoring above a cut-off in STAI-6 anxiety scores warrant additional research. Further research is very likely to change and have an important impact on the confidence on the effect estimate for the outcomes evaluated in the review.

**Table 2:** Summary of findings and quality of the evidence for the outcomes in the review.

	Illustrative comparative risks* **(95% CI)**		**Relative effect (95% CI)**	**No. of participant (studies)**	**Quality of the evidence (GRADE)**	
	**Assumed risk**	**Corresponding risk**				
**Outcomes**	**Control**	**Probiotics**				
Mean EPDS depression scores. Scale from: 0 to 30	The mean EPDS score across control groups was from 6.76 to 9 points (out of 30)	The mean EPDS depression score in the intervention groups was 0.46 lower (2.16 lower−1.25 higher)		545 (2 RCTs)	Risk of bias	⊗⊗◯◯
					Inconsistency	⊗⊗◯◯
					Indirectness	⊗⊗⊗⊗
					Imprecision	⊗⊗◯◯
					Publication bias	⊗⊗⊗◯
% scoring above a cut-off score in the EPDS	19.7 per 100	14.2 per 100 (9.5–20.8)	OR 0.68 (0.43, 1.07)	545 (2 RCTs)	Risk of bias	⊗⊗◯◯
					Inconsistency	⊗⊗⊗◯
					Indirectness	⊗⊗⊗⊗
					Imprecision	⊗⊗⊗◯
					Publication bias	⊗⊗⊗◯
Mean STAI-6 anxiety scores	The mean STAI-6 score across control groups was from 13 to 32.8	The mean STAI-6 anxiety score in the intervention groups was 0.99 lower (1.80–0.18 lower)		543 (2 RCTs)	Risk of bias	⊗⊗◯◯
					Inconsistency	⊗⊗⊗◯
					Indirectness	⊗⊗⊗⊗
					Imprecision	⊗⊗⊗◯
					Publication bias	⊗⊗⊗◯
% scoring above a cut-off score in the STAI-6	22.3 per 100	15.6 per 100 (6.1–34.7)	OR 0.65 (0.23, 1.85)	543 (2 RCTs)	Risk of bias	⊗⊗◯◯
					Inconsistency	⊗⊗⊗◯
					Indirectness	⊗⊗⊗⊗
					Imprecision	⊗⊗⊗◯
					Publication bias	⊗⊗◯◯
Mean SF-36 mental health scores. Scale from 0 to 100	The mean SF-36 mental health score across control groups was from 48.3 to 55.7 (out of 100)	The mean SF-36 mental health score in the intervention groups was 1.09 higher (2.04 lower to 4.22 higher)		224 (2 RCTs)	Risk of bias	⊗⊗◯◯
					Inconsistency	⊗⊗⊗◯
					Indirectness	⊗⊗⊗⊗
					Imprecision	⊗⊗⊗◯
					Publication bias	⊗⊗⊗◯

*CI, Confidence interval; EPDS, Edinburgh Postnatal Depression Scale; OR, Odds ratio; RCT, randomized controlled trial; STAI-6, State Trait Anxiety Inventory 6 item version*.

*The evidence was deemed moderate to low in view of the small number of studies included in the meta-analyses, imprecision of effect estimates, and heterogeneity for some outcomes*.

*⊗⊗⊗⊗ = High; ⊗⊗⊗◯ = Moderate; ⊗⊗◯◯ = Low; ⊗◯◯◯ = Very Low*.

## Discussion

This systematic review and meta-analysis identified early accumulating evidence from three RCTs, involving a total of 713 participants, on the effectiveness of probiotics administered during pregnancy in improving maternal mental health in the perinatal period. Based on 280 women from two RCTs conducted in New Zealand by Dawe et al. (33) and Slykerman et al. (36) the administration of a *Lactobacillus rhamnosus* probiotic (with and without *Bifidobacterium*), initiated at the beginning of the second trimester of gestation, was associated with a statistically significant reduction in the STAI-6 instrument anxiety scores prior to birth and in the postpartum period (MD = −0.99; 95% CI −1.80, −0.18). While not statistically significant, similar trends were observed for reduced depression scores and lower likelihood of women with scores below cut-off values for clinical depression or anxiety. The review did not identify any trials evaluating the effectiveness of prebiotic or synbiotic supplementation, highlighting the need for research in these areas.

The evidence about the effectiveness of probiotics in decreasing mental health symptoms and risks in non-pregnancy populations is conflicted. Recent meta-analyses of RCTs (21, 23) have reported that probiotics reduce depressive symptoms in the general population and that daily probiotic supplementation has beneficial effects on mood, anxiety, and major depressive disorder and cognitive symptoms, with these benefits reported in both healthy and clinically depressed populations (23). However, authors of these reviews report small pooled effects that warrant further investigation. In contrast, another meta-analysis of RCTs concluded that probiotic supplementation does not improve depressive symptoms in the general population (41). A recent systematic review and meta-analysis concluded that probiotic supplementation does not reduce the risk of maternal pregnancy complications (42). This systematic review did not assess maternal mental health outcomes and reviews of mental health did not include studies of pregnant women. Our systematic review and meta-analyses is the first to address this gap in the current literature.

The effectiveness of probiotic intervention is based on a number of factors, such as the mode of therapy, strain of probiotic, and disease indication (43). The Slykerman et al. (36) and Dawe et al. (33) RCTs were similar in their intervention protocol, including capsule administration of the same lactobacillus species that was initiated at a similar time of gestation. Treatment was continued after birth by Slykerman et al. (36) to enable evaluation of postpartum depression and anxiety, whereas it ended before birth in the Dawe et al. (33) RCT and women were evaluated soon after. The Dawe et al. (33) and Mirghafourvand et al. (34) trials both assessed end of pregnancy mental health with the SF-36 instrument, but there were important differences in the time of initiation, duration and formulation of the probiotic. A 4-week yogurt intervention was administered toward the end of the second trimester in the Mirghafourvand et al. (34) trial, at a time when gestational changes in gut microbiota have already commenced toward enrichment with bifidobacteria and lactic acid bacteria (44). Dispensed as capsules, probiotic treatment was initiated by Dawe et al. (33) at the interface of the first and second trimesters, and continued for 18–24 weeks. Even though there were no differences between probiotics and control groups when the mean SF-36 mental health scores from the Dawe et al. (33) and Mirghafourvand et al. (34) trials were combined, individual trial results indicated that participants in the Dawe trial had lower SF-36 mental health scores indicative of more mental health problems (40). Discrepancies in the approach to calculate SF-36 mental health scores [e.g., additive approach (34) vs. norm-based scores (33)] may have also account for systematic differences between the two studies in interpretation of the SF-36 scores. The SF-36 is a quality of life instrument that, although not primarily designed to evaluate depression, it shows high correlation with postpartum depression (45, 46).

To note, different probiotic strains were evaluated in the primary studies: *Lactobacillus rhamnosus* (33, 36), *Bifidobacterium lactis* (33, 34), and *Lactobacillus acidophilous* (34). Since the effects of probiotics are known to be strain-specific (47) due to different mechanisms of action (48), firm recommendations cannot be drawn from this systematic review on which probiotic is more effective to reduce anxiety or depression symptoms during and after pregnancy.

Study population characteristics may have also influenced probiotic effectiveness. Obesity has been linked to altered composition of the gut microbiome during pregnancy (49). The median BMI of pregnant women in the Slykerman et al. (36) trial for the intervention and control groups was 25.1 and 25.9, respectively. Falling in the range of overweight and obesity, the BMI of pregnant women in the intervention and control groups in the Dawe et al. (33) trial was 38.67 and 38.70, respectively. Thus, there is a possibility that in the Dawe et al. (33) trial, the potential beneficial mental health effects of the probiotic intervention were impaired by the baseline microbiota changes in the obese pregnant women. In view of geographic area differences in gut microbial composition (50), the conduct of the Dawe et al. and Slykerman et al. RCTs in the same country (of New Zealand) may ensure a level of homogeneity in baseline gut microbial composition. However, these trials enrolled women with a different ethnicity and socioeconomic profiles, and likely, with dissimilar underlying risk for poor mental health (51) and dysbiosis of pregnancy gut microbiota (52). It is noteworthy then, that despite potential differences in the psychosocial and gut microbial profile of study women across the two RCTs, a benefit was found for a lactobacillus probiotic intervention. Since mental health is influenced by social, economic, and physical environments, future studies should investigate how social adversity influence microbiota-gut-brain communications in pregnancy to affect maternal mental health.

Studies included in this systematic review were RCTs rated at low risk of selection bias; hence, it is likely that randomization allowed a balance between treatment and control groups for known prenatal and perinatal factors (e.g., preterm-birth, vaginal delivery, feeding methods, and socioeconomic and employment status) that may confound the impact of probiotic supplementation during pregnancy on mental health outcomes during and after pregnancy. However, using randomization alone to equally distribute potential confounders between the groups does not automatically protect against selection bias. As allocation concealment (which is another important pre-requisite in RCTs to prevent selection bias) was rated unclear in one of the studies (34), complete avoidance of selection bias introduced by unknown confounders cannot be guaranteed.

This systematic review did not examine potential mechanisms for the probiotic, prebiotic, or symbiotic effects on perinatal mental health symptoms; however, some modes of action investigated in preclinical studies may account for the probiotic effect of lowering anxiety and depression scores identified in this review. Rodent model studies have suggested that probiotics can reduce chronic stress markers (e.g., adrenocorticotropic hormone, corticosterone, adrenaline, and noradrenaline) and attenuate hypothalamic-pituitary-adrenal (HTA) axis responses, which are hyperreactive in depressed patients (53). Others have proposed that probiotics act as modulators of tryptophan and metabolite 5-Hydroxy indoleacetic acid, which are important precursors of critical neurotransmitters implicated in anxiety and depression and known to be synthesized by the gut microbiota (23, 54). Finally, probiotics have been also implicated in a reduction of proinflammatory cytokines (i.e., interleukin-1-beta and interleukin-6) and microglial activation markers that have been shown to be increased in studies evaluating inflammation as one of the explanatory pathways for onset and maintenance of depression (23, 55). Future clinical studies in human exploring these potential mechanisms will allow for appropriate strain selections and perhaps uncover novel strain functions to target the heterogeneous nature of both the gut microbiota composition and the clinical presentation of depressive and anxiety symptoms during the perinatal period.

### Strengths and Limitations

Strengths of our systematic review approach include the use of a comprehensive search strategy in both electronic and gray literature sources and involving of two reviewers in all stages of the review process as strategies to avoid selection bias in the review. The risk of bias and GRADE evaluations provided insightful information about the strengths and weaknesses of this body of evidence. Meta-analyses were conducted using methods that accounted for statistical and clinical heterogeneity across the studies. Our review is limited by the small number of RCTs evaluating the administration of probiotics during pregnancy to reduce mental health problems in the perinatal period and the moderate quality of the body of evidence for the outcomes evaluated in the primary studies.

## Conclusion

The use of probiotics, prebiotics and synbiotic supplementation during pregnancy is an emerging area of research. This systematic review found limited evidence about the effectiveness of probiotics administered during pregnancy to reduce the risk of maternal mental health disorders and highlighted the lack of evidence on prebiotics and synbiotics supplementation to inform their use for similar purposes. Firm clinical recommendations about the use of probiotics, prebiotics, and synbiotics to prevent the occurrence of mental health problems in the perinatal period cannot be based on the current body of evidence about their effectiveness. Finally, it is imperative that future trials of microbiota interventions test probiotic/prebiotic/synbiotic interventions that redress specific dysbioses in pregnancy gut microbiota that arise from poor mental health.

## Data Availability Statement

The original contributions presented in the study are included in the article/Supplementary Materials, further inquiries can be directed to the corresponding author.

## Author Contributions

MO, AK, and JW conceived and designed the study. VD, SL, OS, LD, and MO collected and analyzed the data. VD and MO drafted the manuscript. All authors approved the contents of the manuscript.

## Conflict of Interest

The authors declare that the research was conducted in the absence of any commercial or financial relationships that could be construed as a potential conflict of interest.
