# Supplementary material for: Effectiveness of Probiotic, Prebiotic, and Synbiotic Supplementation to Improve Perinatal Mental Health in Mothers: A Systematic Review and Meta-Analysis
Source: Front Psychiatry. 2021 Apr 22;12:622181. doi: 10.3389/fpsyt.2021.622181 (PMC8100186; doi:10.3389/fpsyt.2021.622181)
Supplement: Supplementary file 1 [file Table_1.docx]

**Supplementary Material 1.** **Electronic Search Strategies**

**1. MEDLINE**

**Ovid MEDLINE(R) ALL 1946 to August 19, 2020**

Date searched: Aug 19, 2020

Results: 470

1. pregnancy/ or prenatal nutritional physiological phenomena/

2. (pregnan* or prenatal or antenatal).tw,kf.

3. 1 or 2

4. prebiotics/ or probiotics/ or synbiotics/

5. (prebiotic* or probiotic* or synbiotic*).mp.

6. 4 or 5

7. exp Lactobacillaceae/

8. exp Bifidobacterium/

9. exp Saccharomyces/

10. exp lactococcus/

11. exp Bacillus/

12. (lactobacill* or bifidobacter* or saccharomyc* or lactococc* or bacillus or pediococc* or leuconostoc*).tw,kf.

13. (lactic acid bacteria or acidophilus or reuteri or lactis or helveticus or longum or plantarum or gasseri or rhamnosus or bifidum or casei or paracasei or infantis or adolescentis or thermophilus or salivarius or animalis or breve or boulardii or butryicum or faecalis or "vsl#3" or "sf68" or Propionibacterium-freundendsreichii).tw,kf.

14. Oligosaccharides/

15. (oligosaccharide* or fructooligosaccharide* or galactooligosaccharide* or xylooligosaccharide*).mp.

16. (dietary fib* adj3 (indigest* or nondigest* or non-digest*)).mp.

17. or/7-16

18. Dietary Supplements/

19. (supplement* not supplementary-material*).tw,kf.

20. Gastrointestinal Agents/

21. (ad or tu or th).fs.

22. or/18-21

23. 3 and (6 or (17 and 22))

24. exp Clinical trial/ or randomized.tw. or placebo.tw. or dt.fs. or randomly.tw. or trial.tw. or groups.tw.

25. 23 and 24

26. limit 25 to animals

27. limit 26 to humans

28. 25 not (26 not 27)

29. 28 not (mouse or mice or rat or rats or sows or pig or pigs or foals).ti.

30. (meningitis or sepsis or vaccin* or gestational diabetes or hypertension or preeclampsia or influenza).ti.

31. 29 not 30

**2. EMBASE**

**Ovid EMBASE(R) ALL 1946 to August 19, 2020**

Date searched: Aug 19, 2020

Results: 1,052

1. pregnancy/ or first trimester pregnancy/ or second trimester pregnancy/ or third trimester pregnancy/

2. (pregnan* or prenatal or antenatal).tw,kw.

3. 1 or 2

4. prebiotic agent/

5. exp probiotic agent/

6. synbiotic agent/

7. (prebiotic* or probiotic* or synbiotic*).mp.

8. or/4-7

9. exp lactobacillaceae/

10. exp Bifidobacterium/

11. exp Saccharomyces/

12. exp lactococcus/

13. exp Bacillus/

14. (lactobacill* or bifidobacter* or saccharomyc* or lactococc* or bacillus or pediococc* or leuconostoc*).tw,kw.

15. (lactic acid bacteria or acidophilus or reuteri or lactis or helveticus or longum or plantarum or gasseri or rhamnosus or bifidum or casei or paracasei or infantis or adolescentis or thermophilus or salivarius or animalis or breve or boulardii or butryicum or faecalis or "vsl#3" or "sf68" or Propionibacterium-freundendsreichii).tw,kw.

16. oligosaccharide/

17. (oligosaccharide* or fructooligosaccharide* or galactooligosaccharide* or xylooligosaccharide*).mp.

18. (dietary fib* adj3 (indigest* or nondigest* or non-digest*)).mp.

19. or/9-18

20. dietary supplement/

21. diet supplementation/

22. (supplement* not supplementary-material*).tw,kw.

23. gastrointestinal agent/

24. dt.fs.

25. or/20-24

26. 3 and (8 or (19 and 25))

27. exp clinical trial/

28. (randomized or placebo).tw. or dt.fs. or randomly.tw. or trial.tw. or groups.tw.

29. 26 and (27 or 28)

30. limit 29 to (animals and animal studies)

31. limit 30 to human

32. 29 not (30 not 31)

33. 32 not (mouse or mice or rat or rats or sows or pig or pigs or foals).ti.

34. 33 not (meningitis or sepsis or vaccin* or gestational diabetes or hypertension or preeclampsia or influenza).ti.

**3. CINAHL**

**CINAHL Plus with Full Text (EBSCO host interface)**

Date searched: Aug 19, 2020

Results: 404

S1 pregnan* or prenatal or antenatal

S2 prebiotic* or probiotic* or synbiotic*

S3 (MH "Lactobacillus+") OR (MH "Pediococcus") OR (MH "Streptococcus Salivarius") OR (MH "Bifidobacterium") OR (MH "Saccharomyces") OR (MH "Bacillus") OR (MH "Oligosaccharides")

S4 lactobacill* or bifidobacter* or saccharomyc* or lactococc* or bacillus or pediococc* or leuconostoc* or lactic-acid-bacteria or acidophilus or reuteri or lactis or helveticus or longum or plantarum or gasseri or rhamnosus or bifidum or casei or paracasei or infantis or adolescentis or thermophilus or salivarius or animalis or breve or boulardii or butryicum or faecalis or "vsl#3" or "sf68" or Propionibacterium-freundendsreichii or oligosaccharide* or fructooligosaccharide* or galactooligosaccharide* or xylooligosaccharide*

S5 (fibre or fiber) N3 (indigest* or nondigest* or non-digest*)

S6 ((MH "Gastrointestinal Agents") OR (MH "Dietary Supplements")) OR (supplement* not supplementary-material*)

S7 (S3 OR S4 OR S5) AND S6

S8 S1 AND (S2 OR S7) NOT TI ( meningitis or sepsis or vaccin* or gestational diabetes or hypertension or preeclampsia or influenza or mouse or mice or rat or rats or sows or pig or pigs or foals)

S9 (MH "Clinical Trials+") OR (randomized or placebo or randomly or trial or groups)

S10 S8 AND S9

**4. Cochrane Central Register of Controlled Trials**

CENTRAL (Trials Tab in Wiley Cochrane Library

Date searched: Aug 19, 2020

Results: 564

#1 [mh ^"pregnancy"] or [mh ^"prenatal nutritional physiological phenomena"]

#2 (pregnan* or prenatal or antenatal):ti,ab,kw

#3 #1 or #2

#4 [mh ^"prebiotics"] or [mh ^"probiotics"] or [mh ^"synbiotics"]

#5 (prebiotic* or probiotic* or synbiotic*):ti,ab,kw

#6 #4 or #5

#7 [mh "Lactobacillaceae"] or [mh "Bifidobacterium"] or [mh "Saccharomyces"] or [mh "lactococcus"] or [mh "Bacillus"]

#8 (lactobacill* or bifidobacter* or saccharomyc* or lactococc* or bacillus or pediococc* or leuconostoc*):ti,ab,kw

#9 ("lactic acid bacteria" or "acidophilus" or "reuteri" or "lactis" or "helveticus" or "longum" or "plantarum" or "gasseri" or "rhamnosus" or "bifidum" or "casei" or "paracasei" or "infantis" or "adolescentis" or "thermophilus" or "salivarius" or "animalis" or "breve" or "boulardii" or "butryicum" or "faecalis" or "sf68" or "Propionibacterium-freundendsreichii"):ti,ab,kw

#10 [mh ^"Oligosaccharides"]

#11 (oligosaccharide* or fructooligosaccharide* or galactooligosaccharide* or xylooligosaccharide*):ti,ab,kw

#12 ((fibre or fiber) NEAR/3 (indigest* or nondigest* or non-digest*)):ti,ab,kw

#13 #7 or #8 or #9 or #10 or #11 or #12

#14 [mh ^"Dietary Supplements"]

#15 (supplement* not "supplementary-material"):ti,ab,kw

#16 [mh ^"Gastrointestinal Agents"]

#17 [mh /AD] OR [mh /TU] or [mh /TH]

#18 #14 or #15 or #16 or #17

#19 #3 AND (#6 OR (#13 AND #18))

#20 (meningitis or sepsis or vaccin* or "gestational diabetes" or hypertension or preeclampsia or influenza):ti

#21 #19 NOT #20

**5. Scopus**

Date searched: Aug 19, 2020

Results: 735

((TITLE-ABS-KEY (pregnan* OR prenatal OR antenatal) AND (TITLE-ABS-KEY (prebiotic* OR probiotic* OR synbiotic*) OR (TITLE-ABS-KEY ( lactobacill* OR bifidobacter* OR saccharomyc* OR lactococc* OR bacillus OR pediococc* OR leuconostoc* OR lactic-acid-bacteria OR acidophilus OR reuteri OR lactis OR helveticus OR longum OR plantarum OR gasseri OR rhamnosus OR bifidum OR casei OR paracasei OR infantis OR adolescentis OR thermophilus OR salivarius OR animalis OR breve OR boulardii OR butryicum OR faecalis OR "sf68" OR propionibacterium-freundendsreichii OR oligosaccharide* OR fructooligosaccharide* OR galactooligosaccharide* OR xylooligosaccharide* ) AND TITLE-ABS-KEY ( supplement* AND NOT supplementary-material* )))) AND (TITLE-ABS-KEY randomized OR placebo OR randomly OR trial OR groups OR trials))) AND NOT (TITLE ( meningitis OR sepsis OR vaccin* OR gestational-diabetes OR hypertension OR preeclampsia OR influenza OR mouse OR mice OR rat OR rats OR sows OR cow OR cows OR pig OR pigs OR foals)

**6. Web of Science**

**Web of Science Core Collection**

Indexes=SCI-EXPANDED, SSCI, A&HCI, CPCI-S, CPCI-SSH, BKCI-S, BKCI-SSH, ESCI, CCR-EXPANDED, IC Timespan=All years

Date searched: Aug 19, 2020

Results: 450

#1 TS=(pregnan* OR prenatal OR antenatal )

#2 TS=( prebiotic* OR probiotic* OR synbiotic* )

#3 TS=(lactobacill* OR bifidobacter* OR saccharomyc* OR lactococc* OR bacillus OR pediococc* OR leuconostoc* OR lactic-acid-bacteria OR acidophilus OR reuteri OR lactis OR helveticus OR longum OR plantarum OR gasseri OR rhamnosus OR bifidum OR casei OR paracasei OR infantis OR adolescentis OR thermophilus OR salivarius OR animalis OR breve OR boulardii OR butryicum OR faecalis OR "sf68" OR propionibacterium-freundendsreichii OR oligosaccharide* OR fructooligosaccharide* OR galactooligosaccharide* OR xylooligosaccharide*) AND TS=(supplement* NOT supplementary-material*)

#4 TS=( randomized OR placebo OR randomly OR trial OR groups OR trials)

#5 TI=( meningitis OR sepsis OR vaccin* OR gestational-diabetes OR hypertension OR preeclampsia OR influenza OR mouse OR mice OR rat OR rats OR sows OR cow OR cows OR pig OR pigs OR foals)

#6 (#1 AND (#2 OR #3) AND #4) NOT #5

**7. BIOSIS**

**Biosis Citation Index (Web of Science Interface)**

Indexes=BCI Timespan=All years

Date searched: Aug 19, 2020

Results: 193

#1 TS=(pregnan* OR prenatal OR antenatal)

#2 TS=( prebiotic* OR probiotic* OR synbiotic*)

#3 TS=(lactobacill* OR bifidobacter* OR saccharomyc* OR lactococc* OR bacillus OR pediococc* OR leuconostoc* OR lactic-acid-bacteria OR acidophilus OR reuteri OR lactis OR helveticus OR longum OR plantarum OR gasseri OR rhamnosus OR bifidum OR casei OR paracasei OR infantis OR adolescentis OR thermophilus OR salivarius OR animalis OR breve OR boulardii OR butryicum OR faecalis OR "sf68" OR propionibacterium-freundendsreichii OR oligosaccharide* OR fructooligosaccharide* OR galactooligosaccharide* OR xylooligosaccharide*) AND TS=(supplement* NOT supplementary-material*)

#4 TS=( randomized OR placebo OR randomly OR trial OR groups OR trials)

#5 TI=( meningitis OR sepsis OR vaccin* OR gestational-diabetes OR hypertension OR preeclampsia OR influenza OR mouse OR mice OR rat OR rats OR sows OR cow OR cows OR pig OR pigs OR foals)

#6 (#1 AND (#2 OR #3) AND #4) NOT #5
